# Experimental Study of HFE 7000 Refrigerant Condensation in Horizontal Pipe Minichannels

**DOI:** 10.3390/ma14226886

**Published:** 2021-11-15

**Authors:** Małgorzata Sikora, Tadeusz Bohdal, Karolina Formela

**Affiliations:** Department of Energy, Koszalin University of Technology, ul. Racławicka 15-17, 75-620 Koszalin, Poland; tadeusz.bohdal@tu.koszalin.pl (T.B.); karolina.formela@s.tu.koszalin.pl (K.F.)

**Keywords:** condensation, heat transfer, pressure drop, pipe minichannels, HFE 7000

## Abstract

This article presents the results obtained from our own experimental investigations on heat exchange and pressure drop during the condensation flow of the HFE 7000 refrigerant in pipe minichannels with an internal diameter of d_i_ = 1.2–2.5 mm. The influence of vapor quality x and the mass flux density G on the two-phase flow pressure drops and heat transfer is presented. The tests were performed for the mass flux density range of G = 110–4700 kg/m^2^s, saturation inlet temperature of T_s_ = 36–43 °C and heat flux density of q = 1 ÷ 20 kW/m^2^. The pressure drop characteristics and heat transfer coefficient as a function of the internal diameter of minichannels are illustrated. The results of experimental research on the heat transfer coefficient and two-phase pressure drop are compared with correlations developed by other authors. The best accuracy has a comparison of experimental study with correlation of Rahman-Kariya-Miyara et al. and Mikielewicz et al.

## 1. Introduction

The literature presents various examples of the analysis of condensation in minichannels in many areas [1]. One such analysis was performed by Bohdal et al. [2], in which the authors focused on studies of high-pressure refrigerants such as R404A, R407C and R410A. The process of condensation was recognized in water-cooled minichannels with an internal diameter of d_i_ = 1.2–2.5 mm in the mass flux density range of G = 150–1280 kg/m^2^s and in air-cooled minichannels with the same internal diameters and a mass flux density in the range of G = 100–1100 kg/m^2^s. The impact of the minichannel internal diameter, vapor quality and mass flux density on the heat transfer coefficient was analyzed. An increase in mass flux density was observed in both cases, which determines the increase in the local heat transfer coefficient. A correlation was also proposed for the determination of heat transfer. A very wide review of correlations for heat transfer during condensation in mini and microchannels was conducted by Awad et al. [3]. They analyzed a wide range of flow and heat parameters for such refrigerants, such as C0_2_ or R744, FC72, R22, R410A and R407C. The condensation process was realized in various shapes and orientations of the channels.

In Bohdal et al. [4], the pressure drop and the heat transfer coefficients were specified during the flow of the R404A, R407C and R410A refrigerants in air-cooled and water-cooled minichannels with diameters of d_i_ = 1.2–2.5 mm in the mass flux density range of G = 100–2200 kg/m^2^s and in minicondensers consisting of two bundles of minichannels with an internal diameter of 0.64 mm and a length of 100 mm. The mass flux density range, in this case, was G = 150–1200 kg/m2s. A reduction in heat transport efficiency in multiports was observed in relation to the single minichannel with the same internal diameter. On the other hand, in studies by Qiu et al. [5], numerical simulations during the condensation of the R290 refrigerant in minichannels with diameters equal to 0.5, 1.0 and 2.0 mm in the mass flux density range of G = 200–500 kg/m^2^s were described. Research clearly points to the dependence of the flow model, the frictional pressure drop and the value of the heat transfer coefficient on the internal diameter of the minichannel. Al-Zaidi et al. [6] studied the condensation of the HFE 7100 refrigerant in rectangular multiports. This phenomenon was studied in multiports made from minichannels with a hydraulic diameter of 0.57 mm in the mass flow density range of G = 50–250 kg/m^2^s. The increase in the local heat transfer coefficient with the increase in mass flux density was confirmed. A decrease in the efficiency of heat transfer with a decrease in vapor quality was observed. The flow at the inlet, middle and outlet of the channel was visualized in order to show the formation of two-phase structures. In the conducted study, the predominant structure was annular flow, which was subsequently transformed into slug flow. As a result of the further progression of condensation in which the mass fraction of the superheated refrigerant decreased, bubble flow was created. Oliwier et al. [7] also noted the pressure drop and heat transfer coefficient for the R407C, R22 and R134a refrigerants. The authors investigated the condensation process in a smooth pipe with an internal diameter of d_i_ = 8.11 mm, a herringbone pipe with a diameter of d_i_ = 8.52 mm and a miniature finned tube with a helix angle of 18° and an internal diameter of d_i_ = 8.94 mm for the mass flux density range of G = 400–800 kg/m^2^s. The pressure drop in the herringbone pipe decreased by approximately 79% compared to that in the smooth tube and by approximately 27% compared to that in the finned tube. In the study of Mikielewicz et al. [8], the condensation of HFE 7100 and HFE 7000 refrigerants was analyzed. Their research focused on the pressure drop in a minichannel with an internal diameter of d_i_ = 2.3 mm in the mass flux density range of G = 240–850 kg/m^2^s. It was confirmed that the pressure drop increases with decreasing saturation temperature and increasing mass flux density. The obtained results were compared with the correlations presented by other authors. However, in [9], Mikielewicz et al. compared the heat transfer coefficients and pressure drop for the phase transformations of boiling and condensation phenomena. They investigated the following refrigerants based on collected literature data: R404a, R600a, R290, R32, R134a and R1234yf. They presented a model of nonadiabatic annular flow for boiling and condensation processes. The modification of the expression describing the surface shear stress was also proposed. Issues concerning the analysis of the process of condensation were also discussed in other studies. In addition to experimental research, mathematical modeling of this process in minichannels is also of great importance. As these articles contain correlations verified by calculations of pressure drop and heat transfer during condensation, it will be important to analyze correlations useful for calculations performed for minichannels. For this purpose, the correlation of Rahman–Kariya–Miyara [10] can be used to calculate the heat transfer coefficient. It was based on experimental investigation results of R134a refrigerant condensation in a horizontal and rectangular multiport consisting of 20 channels with hydraulic diameters from 0.64 to 0.81 mm for the mass flux density range of G = 50–200 kg/m^2^s. A strong dependence of the heat transfer coefficient on mass flux density, saturation temperature, vapor quality and channel geometry was observed. In the correlation, the authors considered the mutual interactions among the forces of inertia, viscosity and surface tension. However, the form of the equation used to calculate the heat transfer coefficient takes the following form:

(1) αth=Rel0.11·Prl0.45·(ppr)0.1·(x1− x)0.09·ϕv2|tt·λld

where the two-phase multiplier of the pressure drop can be determined by using the following dependence.

(2) $$\phi_{v}^{2}=1+{C\chi }_{\mathrm{tt}}+\chi_{\mathrm{tt}}^{2}$$

The Lockhart–Martinelli parameter is defined by using the following equation.

(3) $$\chi_{\mathrm{tt}}={\left(\frac{1-\;x}{x}\right)}^{0.09}\cdot {\left(\frac{p_{v}}{p_{l}}\right)}^{0.5}\cdot {\left(\frac{\mu_{l}}{\mu_{v}}\right)}^{0.1}$$

For a channel with a circular cross section, the C coefficient can be determined by the following dependence:

(4) C=16·x0.35·(1− x)0.25 ·(ppr)0.31·Retp0.09·Wetp0.09

where the Reynolds, Re_tp_, and Weber, We_tp_, numbers take the following forms.

(5) Retp=G·d·(xμv+1− xμl)

(6) $${\mathrm{We}}_{\mathrm{tp}}=\frac{G^{2}\cdot d}{\sigma }\cdot \left(\frac{x}{\rho_{v}}+\frac{1-\;x}{\rho_{l}}\right)$$

The presence of two phases in the flow renders it very difficult to determine pressure drops. During two-phase flow, it is not only the volume fraction of the liquid and vapor phase that changes but also the shape of the interface boundary. Therefore, the decisive factor is the frictional pressure drop, which can be calculated from the correlation of Mikielewicz et al. [8], which was adapted to perform calculations in minichannels by the modification of the Müller-Steinhagen and Heck model. The authors carried out investigations on HFE 7000 and HFE 7100 refrigerant condensations in a minichannel with an internal diameter of d_i_ = 2.3 mm for the vapor quality range G = 240–850 kg/m^2^s. The frictional pressure drop in this model can be calculated from the following equation:

(7) $${\left(\frac{\mathrm{dp}}{\mathrm{dz}}\right)}_{\mathrm{th}}={\left(\frac{\mathrm{dp}}{\mathrm{dz}}\right)}_{\mathrm{lo}}\cdot \Phi_{\mathrm{lo}}^{2}\cdot \mathrm{Con}$$

which was modified by introducing the confinement number Con.

(8) $$\mathrm{Con}=\frac{\sqrt{\frac{\sigma }{g\cdot \left(\rho_{l}-{\;\rho }_{v}\right)}}}{d}$$

The two-phase multiplier can be calculated as follows:

(9) $$\phi^{2}{}_{\mathrm{lo}}=\left(1+2\cdot \left(\frac{1}{f_{1}}-1\right)\cdot x\cdot {\mathrm{Con}}^{m}\right)\cdot {\left(1-\;x\right)}^{\frac{1}{3}}+\frac{1}{f_{1}}\cdot x^{3}$$

where f is a Fanning’s friction factor.

(10)for Re > 2300, f1=(μlμv)0.25·(ρvρl)(11)for Re < 2300, f1=(μlμv)·(ρvρl)

In Equation (9), the m index is −1 for minichannels.

Currently, extensive research on the use of the thermal properties of HFE 7000 refrigerants is carried out not only in condensation. Woodoc et al. [11] investigated the use of this refrigerant in a silicone mini heat exchanger (MECH-X), which is a heat sink reactor with a thickness of 800 μm. The studies were carried out for three mass flux density values, G = 680, 1440 and 3350 kg m^−2^ s^−1^, and a heat flux density up to 10 MW/m^2^. The exchanger was used to cool electronic components, which were heated to 90 °C. Research on HFE 7000 refrigerant boiling was performed by Eraghubia et al. [12]. The process of boiling was carried out in a sapphire tube with an internal diameter of 8 mm and a length of 120 mm. The authors conducted studies at low mass flux density values of G = 50–150 kg/m^2^s and presented visualization results for the HFE 7000 refrigerant boiling phenomenon. Mohamadi et al. [13] investigated heat transfer with the use of a nanofluid that consisted of an HFE 7000 refrigerant and Al_2_O_3_ as well as SiO_2_ nanoparticles. The study was conducted in a horizontal channel with an internal diameter of 3 mm.

Adebayo et al. [14] proposed the use of an HFE 7000 refrigerant in combination with CO_2_ in a cascade refrigeration device. Wang et al. [15] proposed the use of this refrigerant and HFE 7100 as well as the HFE 7500 refrigerant in the organic Rankine cycle. The use of HFE 7000 refrigerants in heat transfer is becoming increasingly common.

The above analysis of literature sources shows that there are few studies on heat transfer and pressure drop during condensation in minichannels concerning new pro-ecological refrigerants. This also applies to the HFE 7000 refrigerant. The developed calculation correlations often do not allow obtaining calculation results that are similar to each other. Therefore, it is difficult to clearly assess which of them should be used to determine the heat transfer coefficients and pressure drop values precisely. The lack of a large number of experimental study results causes the existence of a research gap, which forces the need to conduct further experimental studies at various research stands in order to assess and independently verify the existing computational relationships of various authors. Such activities will make it possible to recommend selected correlations for practical use in the design of mini heat exchangers. The experimental studies of the condensation process of the new HFE 7000 refrigerant conducted by the authors are aimed at determining the parameters of the condensation process in the flow in pipe minichannels, which will constitute a new basis for research materials allowing in depth analysis of the condensation process in minichannels. Since these phenomena have not been fully investigated yet, new research material that can be used to develop new computational relationships and to verify the existing ones should be collected. The experimental tests carried out with high accuracy on an independent laboratory stand allow the objective verification of what has been performed in this paper.

## 2. Test Stand

**Figure 1:**
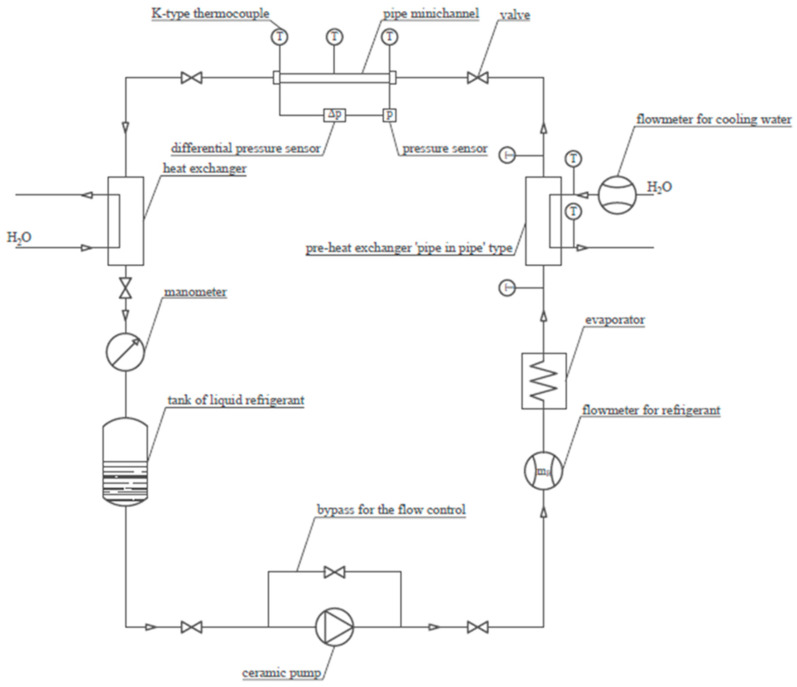
Schematic diagram of the test stand for HFE 7000 refrigerant condensation in minichannels.

**Figure 2:**
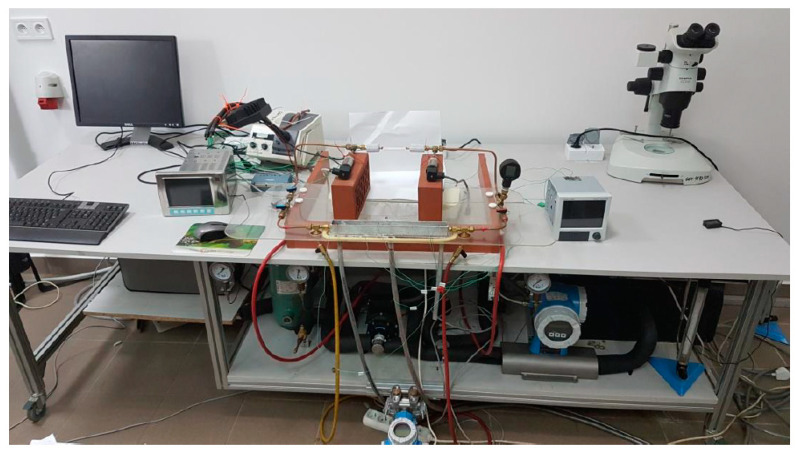
View of test stand.

**Figure 3:**
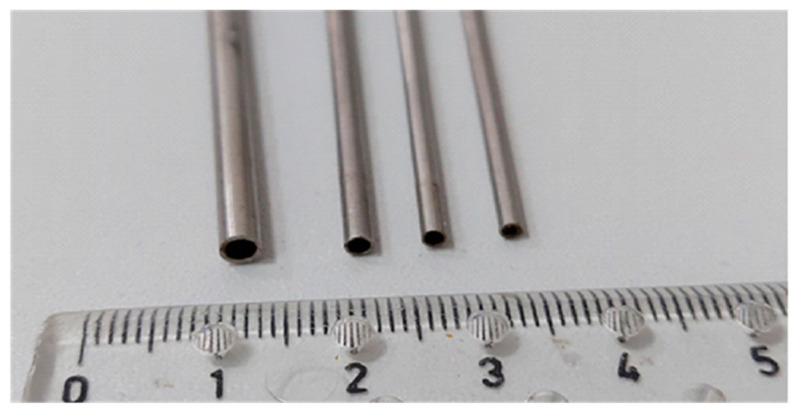
The tested stainless steel minichannels.

Condensation during the flow of the HFE 7000 refrigerant was studied using the test stand illustrated in Figure 1 and Figure 2. In the presented scheme, the liquid refrigerant flows through a ceramic pump and is then pumped into an evaporator. It is an electrically heated heat exchanger. Before the evaporator, a Coriolis mass flow meter was mounted. From the evaporator, the vapor of the refrigerant with a constant temperature flows to the pipe in the pipe heat exchanger. It is a water-cooled heat exchanger that collects the superheat of the refrigerant vapor. Then, the refrigerant flows to the measuring section made of a stainless steel minichannel (L = 250 mm) placed in a water channel. Investigated minichannels are shown in Figure 3. K-type thermocouples are installed in the inlet, middle and outlet of the minichannel and water channel. A pressure sensor is placed at the inlet of the minichannel. A differential pressure sensor is installed at the inlet and outlet of the channel. A heat exchanger is placed after the measuring section in order to subcool the refrigerant.

**Figure 4:**
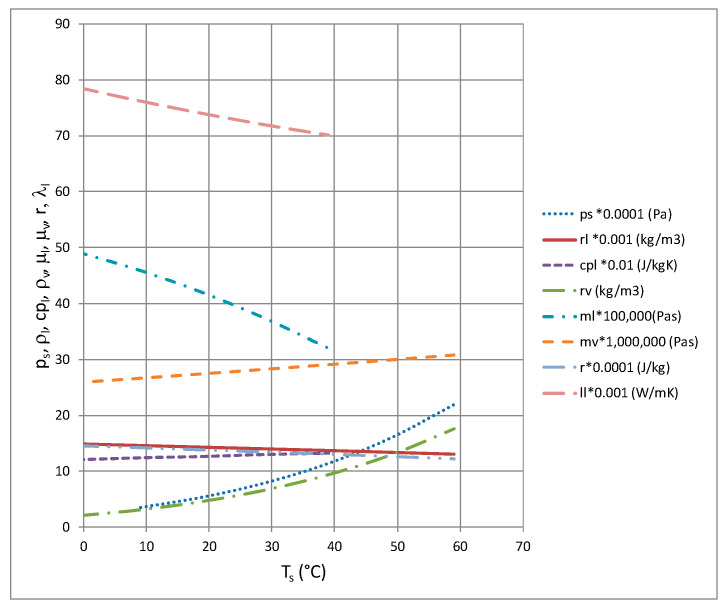
Example properties of HFE 7000 refrigerant [16,17]: saturation pressure p_s_ (ps); heat of evaporation r; specific heat of liquid cp_l_ (cpl); heat conductivity of liquid λ_l_ (ll); liquid density ρ_l_ (rl); vapor density ρ_v_; (rv); liquid viscosity μ_l_ (ml); and vapor viscosity μ_v_ (mv).

The experimental investigations used nonflammable, low-toxicity and noncorrosive substances from the hydrofluoroether group. Examples of the physical properties of HFE 7000 are presented in Table 1. This refrigerant has an ODP parameter equal to zero and a low GWP parameter equal to 530, while the lifetime of this substance is less than 5 years. Figure 4 shows selected physicochemical properties of the HFE 7000 refrigerant belonging to the group of low pressure refrigerants produced by the 3M company [16].

## 3. Experimental Methodology of Investigations

The condensation of the HFE 7000 refrigerant took place inside a water-cooled horizontal minichannel with a length of 250 mm. The test and control equipment used for the test stand allowed direct measurements of the temperature of the cooling water and channel wall as well as the pressure of the refrigerant. The mass flows of the refrigerant and cooling water were measured. Vapor quality was indirectly defined based on the energy balance of the heat exchanger before the inlet to the measuring section. The vapor quality at the inlet was determined based on the energy balance of the preheat exchanger. Using the calculation method, heat flux density was also defined. The method used to determine the heat transfer coefficient of the refrigerant during the condensation process, which was also used in these tests, is described in articles [18,19]. The amount of heat exchanged with the environment during refrigerant condensation in pipe minichannels was determined by using the method developed based on the concept presented in the work by Shin and Kim [20].

For this purpose, two identical measuring sections were made. One section studied the condensation process, and the other section was electrically heated. The same number of thermocouples, which were located at the same distances, was distributed along the length of the channel in both sections and in the water channel. The flow rate and direction of the flow of cooling water were the same in both cases (countercurrent flow). By measuring the parameters of the electric current, the supplied electric power Q_el_ was determined. The parameters of the electric current were adjusted so that the temperature of the channel wall was consistent with the range of temperatures in the condensation process. In accordance with Joule’s law, the heat flux density exchanged by the surfaces of minichannels was determined by means of dependence:

(12) $$q=\frac{{\;\dot{Q}\;}_{\mathrm{el}}}{\pi \cdot d_{h}\cdot L}$$

where L is the length of the minichannel.

The characteristics, q = f(Δt) = f(T_c_ − T_w_), were determined based on the measurement results of electric power, Q_el_, as well as the temperature of the channel wall, T_c,I_, and cooling water, T_w,i_, in subsequent cross sections of the measuring section. This was used to determine the heat flux density q in the measuring section during condensation.

The vapor quality x of the refrigerant at the inlet cross section flowing to the minichannel was determined based on the energy balance of the preheat exchanger. The heat flux donated by the refrigerant can be defined according to the following equation:

(13) $$\dot{Q}={\;\dot{m}}_{R}\cdot c_{R}\cdot \left(T_{R}-{\;T}_{s}\right)+{\;\dot{m}}_{R}\cdot r\cdot \left(1-\;x\right)$$

where c_R_ is the specific heat of the refrigerant, r is the unit heat of the phase transformation of condensation, T_R_ is the temperature of the refrigerant and T_s_ is the saturation temperature under given conditions.

As already mentioned, the minichannel, in which the condensation of the refrigerant was tested, was cooled with water, which completely absorbed the heat of condensation. The use of the Shin and Kim method [20] made it possible to determine the heat flux density on the wall of the cooling channel, and this is the heat transfer coefficient during the condensation process. Such a procedure made it possible to ignore the heat exchange occurring between the cooling water and the environment. Thus, it can be concluded that there was no direct heat loss. The heat of condensation was entirely transferred to the cooling water (100%). However, heat losses to the environment occurred in the preliminary exchanger, where the refrigerant was prepared, which was supplied to the measuring section. These losses were estimated at 8% of the heat output of this exchanger, which translated into the determination of the vapor quality with a relative error of 5%. An analysis of errors in determining the value of the heat transfer coefficient (10%) and pressure drop (8%) was also carried out.

The local value of the vapour quality x_i_ along the minichannel during condensation can be calculated by using the following equation:

(14) $$x_{i}=x_{i-1}-\frac{\left[{\;\dot{m}}_{w}\cdot c_{w}\cdot \left(T_{\mathrm{wi}-1}-{\;T}_{\mathrm{wi}}\right)\right]}{{\;\dot{m}}_{r}\cdot r}$$

where x_i−1_ is the vapor quality in the previous cross-section, x_i_ is the vapor quality in the current cross-section, T_wi−1_ is the temperature of water in the previous cross-section and T_wi_ is the temperature of water in the current cross-section.

These values were used to determine the heat transfer coefficient α of refrigerant, which describes heat transfer during the process of condensation:

(15) $$\alpha_{i}=\frac{q_{i}}{\Delta T_{i}}$$

where ΔT_i_ is the difference between the saturation temperature of refrigerant T_s_ under the given conditions and the temperature of the channel wall in a given cross-section T_ci_. This value was used to determine the thermal-flow characteristics used to model two-phase heat transfer during condensation in minichannels for individual groups of flow structures. Due to the length of the measuring section and, thus, the small change in vapor quality along the length of the minichannel, the average vapor quality x_a_ along the length of the minichannel with L = 250 mm was used.

Experimental investigations were performed using the following range of parameters:

- The internal diameter of the minichannel: $d_{i}=1.2-2.5$ mm;
- The average vapor quality value: $x_{a}=0-1$;
- The mass flux density: G =110−4700 kg/m^2^s;
- The saturation inlet temperature: $T_{s}=36-43\;$°C.

Heat flux depends on the temperature difference of the refrigerant and cooling water and the refrigerant flow rate. Heat flux changes in this investigation was 1–20 kW/m^2^. Bohdal and Kruzel [2] showed that only large changes in the heat flux resulting from different methods of channel cooling (e.g., with water or air) affect the heat transfer coefficient.

The pressure drop along the measuring section was measured using a differential pressure sensor with a Deltabar SPMP transducer with a measuring range of 0 ÷ 1.5 MPa and execution class 0.075. The measuring section was 250 mm long. The pressure difference measured in the measuring section was converted to 1 m of the channel length. A similar methodology was used in the study [21,22,23].

## 4. Experimental Results of Investigations

**Figure 5:**
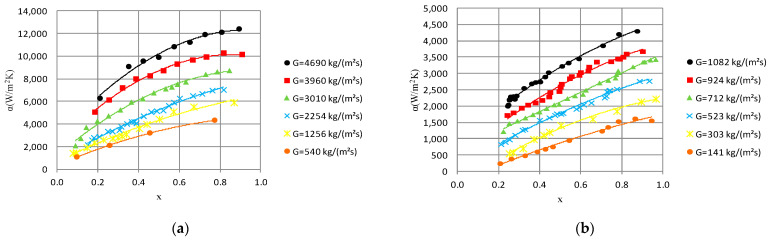
Dependence of heat transfer coefficient on average vapor quality α = f(x_a_) during HFE 7000 condensation in minichannels with internal diameter: (**a**) d_i_ = 1.2 mm, inlet T_s_ = 39 °C; (**b**) d_i_ = 2.5 mm, inlet T_s_ = 42 °C.

Figure 5 presents the selected dependencies of the heat transfer coefficient α on the vapor quality x, which are basic thermal characteristics. The analysis of diagram α = f(x) for a minichannel with an internal diameter of d_i_ = 1.2 and 2.5 mm shows that, as a result of an increase in the average vapor quality value and the mass flux density G, heat transfer intensified. Moreover, under the same condensation conditions, the increase in the internal diameter d_i_ of the minichannel results in a decrease in the heat transfer coefficient α; thus, the efficiency of heat exchange decreases. For example, the increase in the internal diameter from 1.2 mm to 2.5 mm for an average vapor quality value x_a_ = 0.77 and mass flux density G ≈ 536 kg/m^2^s causes a reduction in the heat transfer coefficient from α = 4324 W/m^2^ K to α = 2532 W/m^2^ K. This is a decrease of almost 40%.

**Figure 6:**
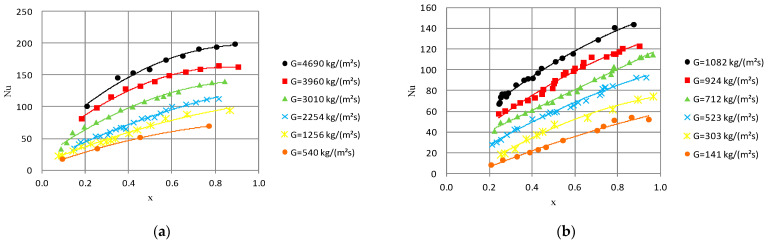
Dependence of Nusselt number on average vapor quality Nu = f(x_a_) for few mass flux densities G during HFE 7000 condensation in minichannels with internal diameter: (**a**) d_i_ = 1.2 mm, inlet T_s_ = 39 °C; (**b**) d_i_ = 2.5 mm, inlet T_s_ = 42 °C.

Heat exchange can also be illustrated as the characteristic of the Nusselt number Nu relative to the average vapor quality value x_a_, which is presented in Figure 6. The Nusselt number, which determines the efficiency of heat transfer between the HFE 7000 refrigerant and the surface of the minichannel wall, depends on the heat transfer coefficient. The increase in the vapor quality value, as well as the mass flux density, determines the increase in the value of the Nusselt number. Moreover, the increase in the internal diameter d_i_ of the minichannel results in an increase in the Nusselt number.

**Figure 7:**
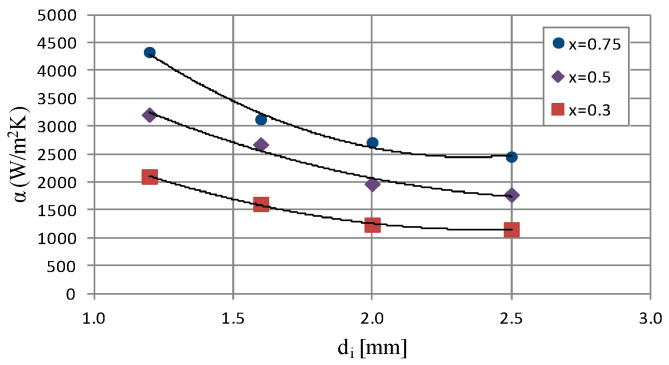
Dependence of heat transfer coefficient on internal diameter of minichannel α = f(d_i_) for G ≈ 519 kg/m^2^s, inlet T_s_ = 39 °C.

Figure 7 presents the characteristics of the heat transfer coefficient α as a function of the internal diameter d_i_ of a single minichannel with a constant mass flux density equal to 519 kg/m^2^s and an average vapor quality value from x_a_ = 0.75 to x_a_ = 0.3. The analysis of the course of the presented graphical dependence shows that there is a noticeable decrease in the efficiency of heat transfer during the condensation process with increasing internal diameter d_i_ of the minichannel. Therefore, the increase in the diameter from 1.2 mm to 2.5 mm causes a nearly two-fold reduction in the heat transfer coefficient from 4324 W/m^2^K to 2452 W/m^2^K.

**Figure 8:**
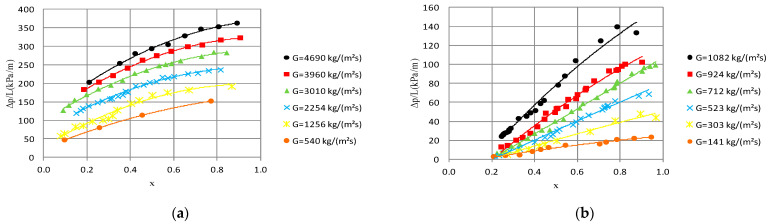
Dependence of pressure drops on average vapor quality (Δp/L) = f(x_a_) for few mass flux densities G during HFE 7000 condensation in minichannels with internal diameter: (**a**) d_i_ = 1.2 mm, inlet T_s_ = 39 °C; (**b**) d_i_ = 2.5 mm, inlet T_s_ = 42 °C.

**Figure 9:**
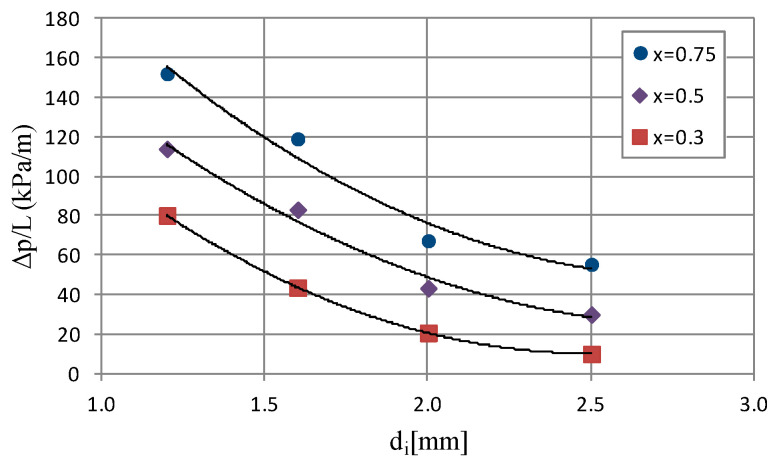
Dependence of pressure drops on internal diameter of minichannel (Δp/L) = f(d_i_) for G ≈ 519 kg/m^2^s and inlet T_s_ = 39 °C.

Figure 8 shows selected dependencies of pressure drop (Δp/L) on average vapor quality value x_a_. The course of the (Δp/L) = f(x_a_) characteristic for an internal diameter range of d_i_ = 1.2–2.5 mm shows that there is a noticeable increase in the values of pressure drop as a result of an increase in the average vapor quality and mass flux density. The internal diameter of the minichannel also has a significant influence. The analysis of the graphical dependence, which is presented in Figure 8, shows that there is a visible increase in pressure drop as a result of the increase in the inner diameter of the minichannel. Therefore, the increase in the internal diameter from 1.2 mm to 2.5 mm for an average vapor quality value x_a_ = 0.77 and a mass flux density G ≈ 536 kg/m^2^s reduces the pressure drop from 152.06 kPa/m to 61.72 kPa/m. Figure 9 presents the dependence of pressure drop (Δp/L) as a function of the internal diameter d_i_ of a single minichannel for a constant mass flux density G = 519 kg/m^2^s and an average vapor quality from x_a_ = 0.75 to x_a_ = 0.75. The analysis of the presented characteristics shows that there is a decrease in pressure drop during the condensation process with increasing internal diameter of the channel. In Figure 7 and Figure 9, the effect of vapor quality on pressure drops and heat transfer coefficient can also be observed, respectively.

**Figure 10:**
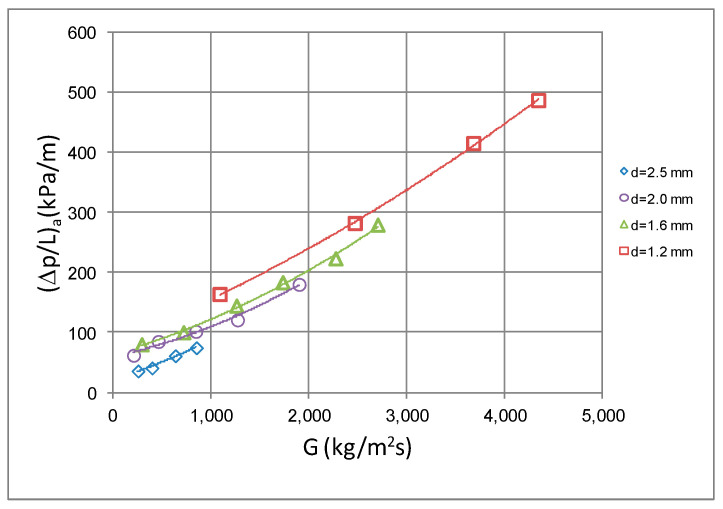
Dependence of pressure drops on mass flux density G for minchannels with internal diameter d_i_ = 2.5, 2.0, 1.6 and 1.2 mm (x ≈ 0.5; T_s_ = 39 °C).

**Figure 11:**
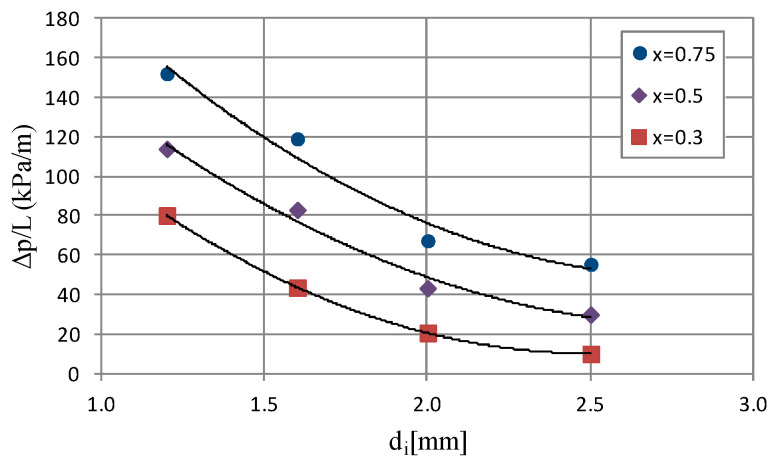
Dependence of heat transfer coefficient on mass flux density G for minchannels with internal diameter: d_i_ = 2.5, 2.0, 1.6 and 1.2 mm (x ≈ 0.5; T_s_ = 39 °C).

Figure 10 shows the dependence of pressure drop (Δp/L) on the mass flux density at a constant average vapor quality value x_a_. Here, the clear influence of the internal diameter and the mass flux density on the pressure drop can also be observed. As observed, the increase in the mass flux density G causes an increase in frictional pressure drop, while the increase in the internal diameter at the same flow results in a decrease. For example, the increase in the mass flux density G from 400 kg/m^2^s to 800 kg/m^2^s causes an increase in pressure drop by more than 40%. Figure 11 presents similar dependencies of heat transfer coefficient α. In this case, the same increase in the mass flux density G results in an increase in the heat transfer coefficient α by more than 30% at constant vapor quality.

**Figure 12:**
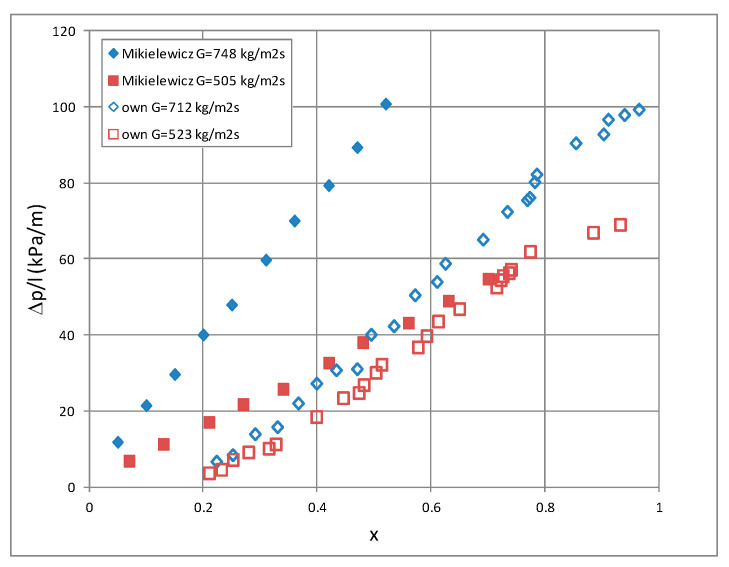
Comparison of own research results with research results of Mikielewicz et al. [9].

**Figure 13:**
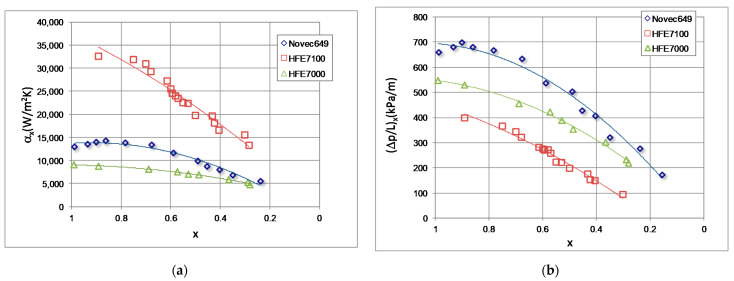
Comparison of our own exemplary results of Novec649, HFE7100 and HFE700 refrigerant condensations in a minichannel with an internal diameter of d_i_ = 1.2 mm with a mass flux density of G = 4500 kg/m^2^s: (**a**) dependence of the heat transfer coefficient on the vapour quality α = f (x); (**b**) dependence of the pressure drop on the vapour quality (Δp/L) = f (x) [24].

Figure 12 shows a comparison of our own research results with the results of the research study conducted by Mikielewicz et al. [9]. Unfortunately, the scope of these studies is not identical, which causes discrepancies in the results. Figure 13 presents a comparison of our own research results on three low-pressure refrigerant condensations in a minichannel with an internal diameter of d_i_ = 1.2 mm. As observed, the highest heat transfer coefficient and the lowest pressure drop in the same conditions have HFE7100, which gives it an advantage over the other two refrigerants (HFE7000 and Novec649) [24].

**Figure 14:**
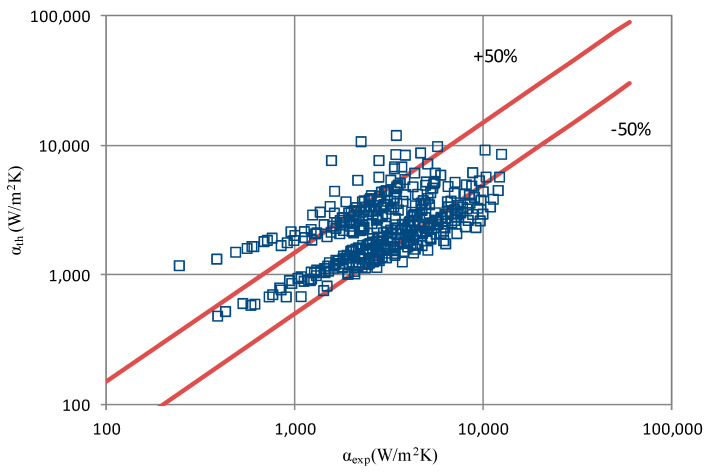
Comparison of the experimental research results on the heat transfer coefficient α with the results of the calculations according to correlation of Rahman–Kariya–Miyara [10] for HFE 7000 refrigerant.

The results of the experimental investigation were compared with the results of calculations according to the correlations of various authors such as the following: Shah et al. [25], Thome et al. [26], Tang [27], Bandhauer [28], Cavallini et al. [29], Rahman-Kariya-Miyara et al. [10], Friedel [30], Chen et al. [31], Garimella et al. [32], Del Col et al. [33] and Mikielewicz et al. [9]. These comparisons are shown in Table 2 and Table 3. In terms of the heat transfer coefficient, the best MAE (65%) accuracy was found for the Rahman–Kariya–Miyara et al. [10] correlation, described in Section 1. Figure 14 presents a graph comparing the experimental heat transfer coefficient α results with the results calculated using the Rahman–Kariya–Miyara et al. correlation (1) for the HFE 7000 refrigerant. As shown in Figure 11, the 80% measurement results obtained during the condensation process of this refrigerant in a channel with an internal diameter in the range of d_i_ = 1.2–2.5 mm have an accuracy of ±50% relative to the calculation results. Considering the fact that this correlation was made for the condensation of the R134a refrigerant in multiports made of rectangular minichannels, a quite satisfactory agreement can be concluded. In particular, the R134a refrigerant belongs to a group of medium-pressure refrigerants, while the HFE 7000 refrigerant is a low-pressure refrigerant. Therefore, the suggested correlation of Rahman–Kariya–Miyara et al. can be roughly used to calculate the heat transfer coefficient during the condensation of the HFE 7000 refrigerant in minichannels. Obviously, there is a need to look for dependencies that will allow the heat transfer coefficient to be calculated with greater accuracy during the condensation of low-pressure refrigerants in minichannels.

**Figure 15:**
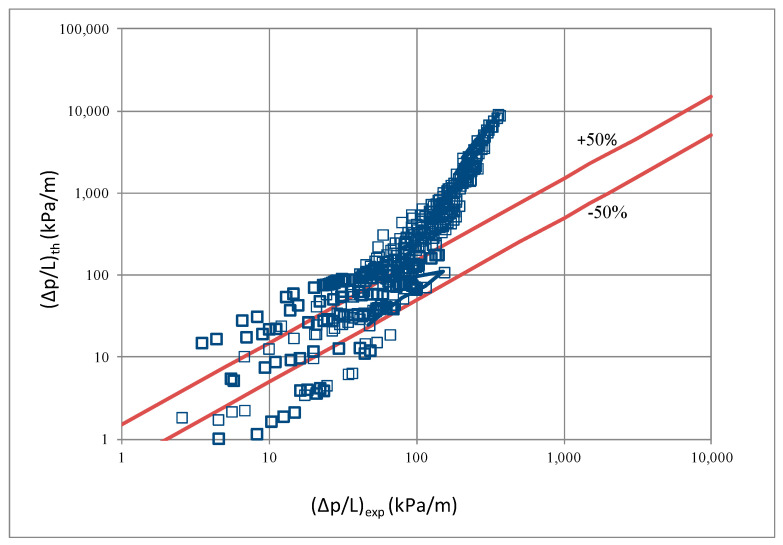
Comparison of the experimental research results on the pressure drops (Δp/L) with the results of the calculations according to correlation of Mikielewicz et al. [9] for HFE 7000 refrigerant.

In terms of pressure drop, the experimental results with the best accuracy and agreement with the calculated results were obtained with the correlation of Mikielewicz et al. [9] (MAE 79%). Figure 15 presents a graph comparing experimental pressure drops (Δp/L) with the results calculated by this correlation according to Equation (7). From the analysis of the comparison (Δp/L)_th_ = f((Δp/L)_exp_) for an internal diameter of d_i_ = 1.2–2.5 mm, it can be observed that the convergence of the experimental results of the thermal and flow investigation during the two-phase condensation transformation in the flow of a refrigerant from the hydrofluoroether group is ±50% for approximately 70% of the points. Considering that the correlation has been modified for the HFE 7000 and HFE 7100 refrigerants, this level of discrepancy is not fully satisfactory. This may be because Mikielewicz et al. [9] verified the correlation for the mass flux density range of G = 240–850 kg/m^2^s, while the results of our own investigations concern a wider range of mass flux densities, G = 110–4700 kg/m^2^s.

## 5. Conclusions

The new low pressure refrigerants are now an attractive alternative to old refrigerants due to their low ODP and GWP coefficients and low operating pressure, which makes them easy to use. They are also non-conductive liquids, which allows them to be used to cool electronics. The conducted investigations confirmed that the process of HFE 7000 refrigerant condensation in pipe minichannels is similar to that of other refrigerants. However, the values of the heat transfer coefficient and pressure drop depend on their physical and chemical properties. The mass flux density G, the vapor quality x and the size of the internal diameter of the minichannel also play an important role. As the heat flux density q on the main-channel wall changed insignificantly, the influence of this change on the values of heat transfer coefficients and pressure drop was negligibly small. This confirmed the observations of other authors described in [2]. As demonstrated in [2] only a large change in the value of q (several dozen times) causes a change in the intensity of the condensation process and a change in the coefficients describing this process. Several detailed conclusions were drawn from the obtained results of the experimental research carried out:

(1) The average vapor quality x_a_, as well as the mass flux density G, has a significant influence on the heat transfer coefficient α change. The dimension of the internal diameter of the minichannel d_i_ also has a meaningful impact on the efficiency of heat transfer. Therefore, the increase in the diameter of the channel from 1.2 mm to 2.5 mm for an average vapor quality of x_a_ = 0.77 and a mass flux density of G ≈ 536 kg/m^2^s results in a reduction in the heat transfer coefficient by almost 40%.
(2) The Nusselt number, Nu, is determined not only by the average vapor quality xa but also by mass flux density G. Moreover, the increase in the dimensionless Nusselt number was observed with the increase in the internal diameter d_i_ of a single channel. As a result, the increase in the internal diameter from 1.2 mm to 2.5 mm for an average vapor quality of x_a_ = 0.77 and a mass flux density of G ≈ 536 kg/m^2^s causes the Nusselt number to increase by almost 20%.
(3) The average vapor quality, x_a_, and mass flux density, G, have significant influences on pressure drop (Δp/L) changes. The parameter that causes the change in the pressure drop is also the internal diameter d_i_ of the minichannel. Therefore, as a result of increasing the internal diameter from 1.2 mm to 2.5 mm for an average vapor quality of x_a_ = 0.77 and mass flux density of G ≈ 536 kg/m^2^s, the value of pressure drop is reduced by almost 60%.
(4) The results of the experimental investigations were compared with the results calculated using the correlation of several authors, including that developed by Rahman-Kariya-Miyara et al. [10] for heat transfer and the correlation developed by Mikielewicz et al. [9] for pressure drop, for which the best compliance was obtained. In both cases, the compliance range was ±50% for approximately 70–80% of the measurement points. This means that there is a need to look for dependencies describing heat transfer and the pressure drop for the condensation of low pressure and low GWP refrigerants in pipe minichannels.

## Figures and Tables

**Table 1:** Physical properties of HFE 7000 refrigerant for working temperature 25 °C [17].

Molecular formula	C_3_H_7_OCH_3_
Molecular weight (g/mol)	200
Boiling point (°C)	34
Freeze point (°C)	−122.5
Liquid density (kg/m^3^)	1400
Critical density (kg/m^3^)	553
Critical pressure (MPa)	2.48
Critical temperature (°C)	165
Dielectric Constant	7.4
Latent heat of vaporization (kJ/kg)	142
Specific heat (J/(kg·K))	1300
Thermal Conductivity (W/(m·K))	0.075
Vapour pressure (kPa)	64.6

**Table 2:** Comparison of heat transfer investigation results with the results of calculations according to the correlations of various authors [10,21,22,23,24,25].

Corelation	
Shah	Thome
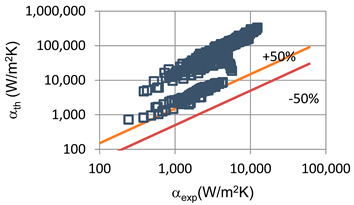	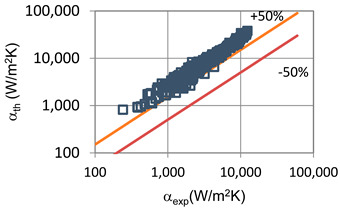
Bendhauer	Cavallini
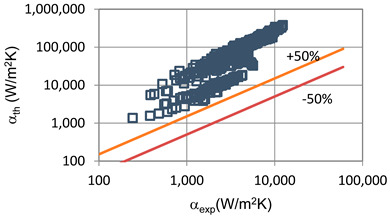	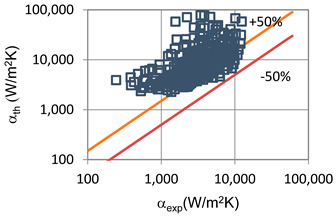
Tang	Rahman
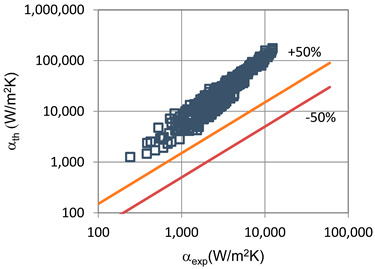	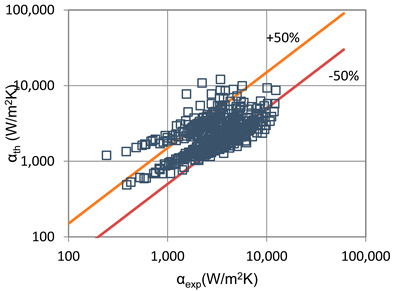

**Table 3:** Comparison of the pressure drop investigation results with the results of calculations according to the correlations of various authors [9,26,27,28,29].

Corelations	
Friedel	Chen
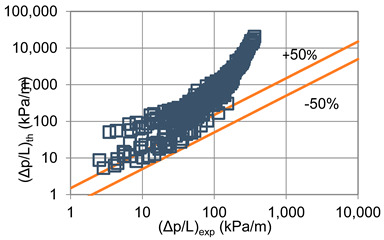	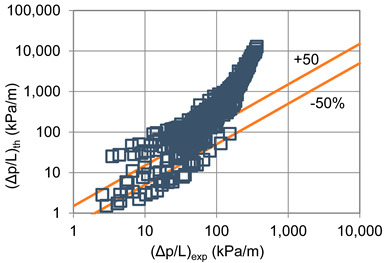
Garimella	Del Col
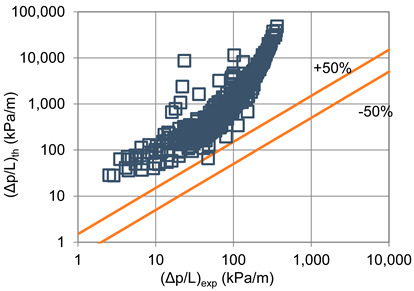	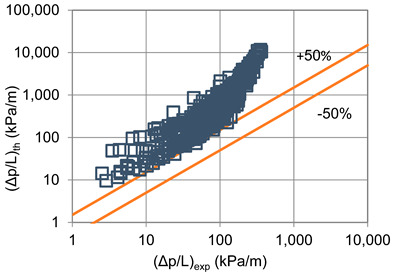
Cavallini	Mikielewicz
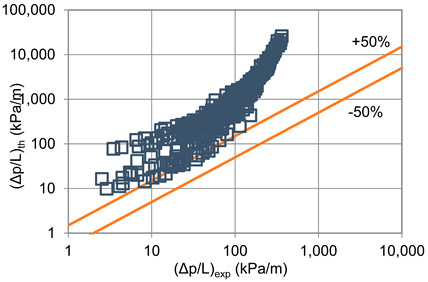	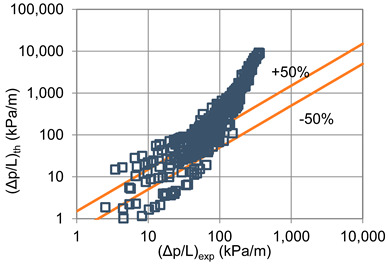

## Data Availability

The data presented in this study are available on request from the corresponding author.

## Nomenclature

C	constant
Con	confinement number
d	diameter, m
dp/dz	frictional pressure drop
f	friction factor
g	acceleration due to gravity, m/s^2^
G	mass flux density, kg/m^2^s
k	thermal conductivity, W/m ·K
L	length, m
ṁ	mass flow rate, kg/s
Nu	Nusselt number
p	pressure, Pa
Pr	Prandtl number
Q	heat flux, W
q	heat flux density, W/m^2^
Re	Reynolds number
T	temperature °C
We	Weber number
x	vapour quality
**Greek**	
α	heat transfer coefficient, W/m^2^ K
μ	dynamic viscosity, Pa·s
ρ	density, kg/m^3^
σ	surface tension, N/m
Φ^2^	two-phase multiplier
Χ_tt_	dimensionless two-phase flow multiplier Martinelli
**Subscripts**	
a	average,
c	channel wall
exp	experimental
h	hydraulic
i	internal
l	liquid
lo	liquid only
R	refrigerant
r	reduced
S	dew point value
v	vapor
th	theoretical
tp	two-phase
w	water
